# Electricity in Uterine Diseases

**Published:** 1885-08

**Authors:** S. F. Starley

**Affiliations:** Ex-Pres. Tex. State Med. Assn., Tyler, Texas


DANIEL'S

### Medical ★ Journal,

PUBLISHED MONTHLY AT

AUSTIN, TEXAS.

Vol. 1.] AUGUST, 1885. [No. 2.

"*Scribimus indocti, doctique!*"

### Original Articles.

CONTRIBUTED EXCLUSIVELY TO THIS JOURNAL.

*The Articles in this Department are accepted, and published with the understanding that we are not responsible for, nor do we indorse the views and opinions of the writers, by so doing.*

### ELECTRICITY IN UTERINE DISEASES.

*By S. F. Starley, M. D., Tyler, Texas.*

For Daniel's Medical Journal.

OF all the morbid conditions to which this most valuable therapeutical agent has been applied, there are none, perhaps, in which its beneficial effects are more strikingly displayed than in the class of cases properly included under the general term "uterine disease." The writer has, for a number of years, given special attention to the use of electricity in the treatment of this, and of other forms of disease, and can testify from actual experience that he has found nothing more helpful in the successful treatment of uterine troubles generally, than the judicious employment of electricity.

In a large proportion of the cases we are called upon to treat, we find, not only that the patient is suffering from some inflammatory engorgement of the uterus, but frequently also from hyperplasia and sub-involution of the organ as well. Now, in this state of things, I know of no equally effective means of getting a start towards breaking up the morbid condition, to that of the mild employment of the electrolytic action of the galvanic current, applied once every second or third day for from 10 to 15 minutes. When we remember that this effect of the constant current is to produce a chemical disturbance of the animal tissues through which it passes, the oxygen and acids going towards the positive, and the hydrogen and alkaline constituents toward the negative pole, we see at once that we have thus produced the most favorable condition for the absorption of the hyperplastic material, not only by the breaking up of its elemental combinations, but by the stimulating effect of electricity over the absorbent vessels.

In most cases of uterine diseases, we find the patient suffering from painful neuralgia, and other disturbances of the nervous system; and here we have in electricity, perhaps, the most potent and durable remedy that we can possibly bring to the relief of our patients. Then there is the well known *nerve tire* and physical, as well as mental, exhaustion, that so constantly attends womb troubles of almost every kind. Such cases require tonics, and while our Materia Medica supplies us with so great a number of valuable remedies of this class, there are none, perhaps, so prompt in their effects, so general in their applicability, and so encouragingly satisfying to our patients, as Faradization properly and perseveringly applied. It at once imparts to the patient a sense of renewed strength of both body and mind. It restores contractive energy to relaxed muscles and ligaments, and aids no little in the restoration of misplaced organs to their normal position; it stimulates and hastens the circulation, and tends powerfully to promote the normal distribution of blood to the different organs of the body, and especially so of the viscera. Its power to promote healthy nutrition is shown by its well known tendency to increase the growth and strength of muscles and of the general increase of body weight, which takes place in persons who are treated by the method of general Faradization for any considerable length of time.

These well known effects of electricity compel us to place it in the very front rank of our resources in the treatment of the class of cases we are considering, and whatever other measures of treatment we may find it necessary to employ, be they medicinal or surgical, we must look to electricity for one of our greatest and most reliable aids in the accomplishment of our main object, i. e., the cure of patients who apply to us for treatment in the multifarious forms of uterine disease.

We will not now encroach upon the valuable space given us by enlarging upon our special methods of employing electricity in certain cases, but will merely state that some of our best results have been obtained by methods of application somewhat different from those laid down in standard works on medical and surgical electricity, though, of course, embracing the same principles of action.

Such of these details as seem to us of practical importance, we hope to communicate, at an early day, through the pages of this Journal.

